# Prevention of Initial Periodontitis Is an Investment in the Future

**DOI:** 10.3390/diagnostics14171850

**Published:** 2024-08-24

**Authors:** Anna Maria Heikkinen, Teija Raivisto, Ismo Tapani Räisänen, Timo Sorsa

**Affiliations:** 1 Faculty of Medicine and Health Technology, University of Tampere, 33100 Tampere, Finland; 2 Wellbeing Services County of Pirkanmaa, 33400 Tampere, Finland; 3 Department of Oral and Maxillofacial Diseases and Public Health, University of Helsinki and Helsinki University Hospital, 00290 Helsinki, Finland; 4 Department of Dental Medicine, Karolinska Institutet, 171 77 Stockholm, Sweden

**Keywords:** adolescent, periodontitis, smoking, Matrix metalloproteinases, gingivitis, biofilms, dental calculus

## Abstract

Background: Periodontal tissue damage is mainly caused by the active form of collagenolytic matrix metalloproteinase-8, aMMP-8, the concentration of which in the mouth rinse can be measured with a mouth rinse chairside-test. The mouth rinse chair side test can be used to identify adolescents with a risk of periodontitis. Methods: The data were collected at the Kotka Health Centre (2004–2005, N = 501 and 2014–2015, N = 47) and at the Hämeenlinna Health Centre (2017–2018, N = 125) consisting of adolescents aged 14–17. They underwent a complete periodontal examination, and some were subjected to the aMMP-8-test. Results: We identified bacterial plaques in combination with increased bleeding on probing (BOP), elevated aMMP-8 concentration, smoking and male sex as the main risk factors for initial periodontitis. Approximately 10% of adolescents had subclinical periodontitis, they were not periodontally healthy, but also not sick. They may not develop periodontitis, but they are at the risk. The aMMP-8 test positivity had a stronger association with initial periodontitis than BOP. Conclusions: In addition to identifying risk factors, these adolescents need individual prevention and, if necessary, early treatment. For the periodontal health biomarker aMMP-8, test negativity ([-], ≤20 ng/mL) should be sought.

## 1. Introduction

Periodontitis is a multifactorial biofilm-induced inflammatory disease [1]. It is the sixth most common chronic infectious disease in the world [2]. In Finland, 75% of adults suffer from periodontitis [3] and 10–15% of adolescents suffer from the early stages of periodontitis [4,5]. Aggressive and rapidly progressive periodontitis in adolescents [6,7], which can be detected by radiological bone changes and involves certain pathogens such as *Aggregatibacter actinomycetemcomitans* (*A.a.*) [8,9], has been identified. However, its prevalence is only about 0.1% [10]. It should be noted that periodontitis can begin in early adolescence and studies suggest that susceptibility to periodontitis follows into adulthood [11,12,13,14]. Many systemic diseases, such as diabetes [15,16,17,18] and cardiovascular diseases [19,20], are associated with periodontitis. Diabetes and periodontitis seem to be bidirectionally linked [21]. Furthermore, smoking is an essential risk factor to periodontitis [22].

Periodontal diseases are traditionally diagnosed in conjunction with clinical and radiological examination. However, this does not provide information on the current state of periodontium and the current potential activity of tissue destruction, but on the already occurred tissue damage. In the early stages of periodontitis (the so-called subclinical preperiodontal condition), the clinical signs of the disease are usually not yet visible and not clinically measurable [4,23,24].

In the new classification of periodontitis [6,25], gingivitis is defined as bleeding on probing (BOP) > 10% and initial periodontitis (stage I) with BOP > 10%, pocket depth (PD) of 4 mm, loss of fixation level (CAL) of 1–2 mm, and bone loss up to the coronal third of the roots [17].

The risk of periodontitis and the development of periodontitis can be determined by measuring the concentration of inflammatory markers in oral fluids [26]. These proinflammatory enzyme concentrations increase in the oral fluid even before clinical signs of inflammation or tissue destruction appear. Matrix metalloproteinases (MMPs) are a group of genetically distinct but structurally related proteolytic host-derived enzymes whose number and activation increase in disease conditions. Matrix metalloproteinase-8 (MMP-8) is the major collagenase in periodontitis [27] and its active form aMMP-8 [19,20,21,24] is the most significant cause of periodontal tissue destruction. The prevalence and level of aMMP-8 can be detected and measured with the aMMP-8 chairside test, where either a visually positive (+) or negative (−) result in the test stick can be read quantitatively by a reader within 5 min [23,26,28].

Periodontal disease has been mostly studied among adults, who already have the disease. More seldom have been studied adolescents and young adults that are the age group that could benefit the most from disease prevention before periodontitis initiates. The identification of periodontitis, especially in adolescents, will probably require a new approach and tools, also from the perspective of prevention. The aim of our studies on young people [14,23,29] was to clarify the prevalence, risk factors and diagnostics of periodontitis in adolescents and to create an understanding of to whom and how periodontal disease prevention should be targeted.

## 2. Materials and Methods

**Research data for adolescents**

The first research and second research data were collected at the Kotka Health Centre in 2004–2005 (N = 501) and 2014–2015 (N = 47), respectively and the third at the Hämeenlinna Health Centre in 2017–2018 (N = 125) as part of our three doctoral dissertation research [4,23,29]. The data for Kotka consisted of persons aged 15 to 17 and that of Hämeenlinna for those aged 14 to 15. The research plans were approved by the City of Hämeenlinna, the HUS Ethics Committee (HUS 1770/2017) and the ethics committees of Kymenlaakso Central Hospital and HUS (HUS 260/13/03/00/13). Inclusion criteria for patient recruitment were the following: age of 15 to 17 years (Kotka studies) or 14 to 15 years (Hämeenlinna study) and willing to participate with a consent to the study participation from the adolescent and/or parents. The age of con-sent is 15 years in Finland. Thus, all participants in the Kotka Health Centre studies were eligible to provide a written informed consent and parental consent was required only in the Hämeenlinna Health Centre study by the institutional review board. Most of the participants were healthy and only a few had general diseases: allergies (N = 10), endocrinological diseases (N = 3), respiratory diseases (N = 7), and skin diseases (N = 4). Participants included had not received systemic antibiotics or periodontal therapy in the previous 12 months.

### 2.1. Kotka Health Centre Studies

In the first age cohort in the city of Kotka (2004–2005, N = 501) in Finland, data on the oral health of adolescents were collected, each adolescent underwent a periodontal examination, in which the depth of the deep periodontal pocket was measured (pocket depth = PD, at least 4 mm depths, on four tooth surfaces), plaque index was determined (visible plaque index = VPI, on the surfaces of index teeth), bleeding on probing was recorded (=BOP, on all six tooth surfaces), dental calculus amount was recorded (=root calculus = RC, on the surfaces of index teeth) and attachment loss (attachment loss = AL) was determined from bite-wing X-rays. In this study, plaque samples were collected from gingival pockets for some of the subjects (N = 264) for practical reasons.

In the second age cohort (2014–2015), 120 subjects were invited, but only 47 of them gave their consent to the study. Only those adolescents who would otherwise have been invited to the oral examination according to the individual examination interval were invited to the study. In the second study, a similar periodontal study was performed in the same way as in the previous age cohort, but prior to the clinical study, the subjects underwent an aMMP-8 mouth rinse chairside test (N = 47). It`s result was known to the author of the periodontal examination of the patient only after the end of the oral examination of the patient. Adolescents were normally treated after the study [26].

### 2.2. Hämeenlinna Health Centre Study

In the city of Hämeenlinna in Finland, N = 190 8th class students (a cohort) were invited, and 125 students agreed to the study, 65 students did not want to participate in this study or did not come to the oral health reception at all. The participants were randomized into the experimental group (N = 70; 29 girls and 41 boys) and the control group (N = 55; 27 girls and 28 boys) to minimize the potential for bias in the assignment process. Participants in the control group were treated according to standard treatment protocol. A mouth rinse sample was collected from the experimental group to analyze the aMMP-8 concentration using the aMMP-8 mouth rinse chairside test. In Hämeenlinna, a dental hygienist performed a health examination, based on which the dental hygienists sent the adolescents to a dentist for oral examination, if necessary.

The PD (≥4 mm) and BOP were measured on the six surfaces of each tooth, RC and VPI on the index teeth. The aMMP-8 chairside test was performed prior to the clinical trial. Mouth rinse samples were collected for analysis later (samples were frozen). The aMMP-8 test result was recorded as either positive (++), weak positive (+), or negative (−).

In the analysis, both positive results were combined. In Hämeenlinna, aMMP-8 test positive patients (N = 24) were given individual self-care health guidance and, if necessary, professional removal of bacterial plaques and tartar (scaling and root planning) was carried out. The test was repeated every 4 months until the test result was negative [23].

### 2.3. Health Behavior Questionnaires

Adolescents in both cities’ of Hämeenlinna and Kotka cohorts filled out a questionnaire with questions about oral care care habits, snacking, use of tobacco and nicotine products, alcohol and drugs. The questionnaires were sent home with the appointment time. The form was sent in advance to adolescents to fill out at home and reviewed with an oral health care professional in connection with the oral health care examination [4,23].

### 2.4. Information and Treatment of Adolescents

All subjects received individual instructions on maintaining good oral hygiene (brushing and interdental cleaning). They received health guidance on how to stop using nicotine products, alcohol use and a healthy diet. The dentist provided health guidance in the Kotka Health Centre study and the dental hygienist in the Hämeenlinna Health Centre study. Periodontal treatment included professional removal of bacterial plaques and tartar (scaling and root planing). The dental hygienist showed the adolescents of Hämeenlinna the result of the aMMP-8 mouth rinse chairside test result, motivating the adolescent to take care of their oral health. At the same time, he/she also discussed about periodontal diseases, their effects and prevention. The follow-up period for test positivity was four months [4,23].

### 2.5. Statistical Analysis

**Figure 1:**
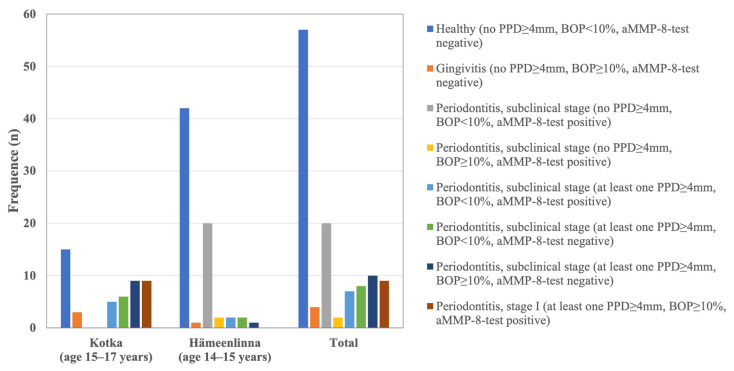
Division of young people in Kotka and Hämeenlinna into healthy, gingivitis, subclinical phase of periodontitis and stage I of periodontitis. Modified from Heikkinen et al. (2022) [24] under the terms and conditions of the Creative Commons Attribution (CC BY) license https://creativecommons.org/licenses/by/4.0/, accessed on 25 July 2024.

The Figure 1 was constructed with Microsoft^®^ Excel^®^ for Mac (Version 16.78) by modifying the data from Heikkinen et al. (2022) [24] under the terms and conditions of the Creative Commons Attribution (CC BY) license https://creativecommons.org/licenses/by/4.0/, accessed on 25 July 2024.

## 3. Results

Based on the results of Kotka’s 1st cohort study (Anna Maria Heikkinen’s doctoral dissertation [4]), initial periodontitis and related clinical signs were detected in 10–15% of the adolescents studied. Smoking appeared to be a significant etiological risk factor for oral health, considering both clinical indices and periodontal inflammatory marker, such as aMMP-8. The amount and duration of smoking (tobacco years) intensified the adverse effects of smoking. The periodontopathogenic bacteria *Prevotella nigrescens* (*P.n.*), *Prevotella intermedia* (*P.i.*), *Tannerella forsythia* (*T.f.*) and *Treponema denticola* (*T.d.*), detected by polymerase chain reaction analysis (PCR), were observed more frequently in young smokers than in non-smokers. Preliminary studies found that adolescents who received smoking cessation intervention and health guidance from an oral health professional quit smoking compared to those who did not. The study also found that some adolescents are strongly dependent on nicotine. There were no adolescents with using or experimented with drugs, and with alcohol the prevalence was below 10%.

In Kotka’s 2nd cohort study, 14 (30%) of the 47 subjects tested positive [26]. Of the 47 participants in the study, 29 adolescents had at least one 4 mm PD and 22 had at least two 4 mm PDs. Of these, 14 adolescents tested positive for the aMMP-8 test. There was a total of 15 adolescents who tested negative for aMMP-8 test and had at least one 4 mm PD. In addition, of those with at least two 4 mm PDs, 8 adolescents were aMMP-8 test negative. It should be noted, however, that all adolescents with at least six 4 mm PDs tested positive for aMMP-8 [26].

The purpose of the study was to determine the suitability of the aMMP-8 mouth rinse chairside test to reliably detect periodontitis in adolescents in its early phase. The aMMP-8 test found nearly 80% of people with initial periodontitis and hardly diagnosed healthy people diseased (96.7%) i.e., false positives. In the Hämeenlinna study (Teija Raivisto’s doctoral dissertation [23]), 24 (34.3%) of the 70 subjects in the test group were test positive. Among those without PDs, 43 were test negative and 22 were test positive. Those with at least one 4 mm PD had three test negative and two tested positive. With enhanced and targeted individual prevention, the positive result of the aMMP-8 test turned negative during the 4-month follow-up period [23].

In the Hämeenlinna cohort data, about 30% had no healthy sextants at all (CPI = 0). Girls had more healthy sextants than boys. A positive aMMP-8 test result came only to those who needed periodontitis treatment, i.e., the CPI index was 1, 2 or 3. Plaque and tartar indices (VPI and RC %) were significantly lower, especially in adolescents who tested negative after one treatment period. Thus, the test found adolescents with poor oral hygiene who were at risk of developing initial periodontitis without yet showing clinical signs of the disease.

When the data from Kotka and Hämeenlinna cohorts were combined (N = 117) (see Figure 1) [24], it was found that there were nine adolescents with at least one 4 mm PD, a positive aMMP-8 test and BOP of at least 10%. In addition, seven adolescents had at least one 4 mm PD, tested positive for aMMP-8 and had a BOP of less than 10%. Taken together, 16/117 × 100% = 14% of young people who can be found to have initial periodontitis. In addition, a total of two adolescents with BOP ≥ 10% and 20 test positives with no clinical findings were found to be aMMP-8 positive [24].

Based on the dissertation of Ismo Räisänen [29], the main risk factors for initial periodontitis were elevated aMMP-8 concentrations, tartar especially in combination with BOP measured in connection with elevated PD measurement, smoking years and male gender. Alcohol and drug use was not shown to be significant in this study.

Obesity and underweight were also associated with initial periodontitis, and in addition, periodontopathogenic bacteria, *Treponema denticola* (*T.d.*) and *Tannerella forsythia* (*T.f.*), could be seen against the background of the risk of periodontitis that begins in bacterial biofilm dysbiosis. In addition, the dissertation showed that the aMMP-8 mouth rinse chairside test had a clearly stronger association with initial periodontitis than BOP. The level of oral hygiene (plaque and tartar) had a much more significant effect on BOP than, for example, PDs or aMMP-8 concentration. The dissertation also found that the aMMP-8 concentration in the mouth rinse compared to saliva is more strongly associated with initial periodontitis.

## 4. Discussion

We found that for all three of our datasets (N = 673, 14–17 years old), approximately 10% were able to detect clinical findings indicating initial periodontitis and/or risk of developing it. Nazir et al. [5] report in their article on the global prevalence of periodontitis in different ages that approximately 9% of teenagers (15–19 years old) were reported to have 4–5 mm PDs and thus initial periodontitis or even stage I periodontitis [5]. In addition, Nazir et al. [5] found that approximately 20% of adolescents have bleeding gums and about 50% have tartar. In the Finnish data of this review, tartar was highlighted as the main risk factors for periodontitis, especially in combination with bleeding on probing (BOP), elevated aMMP-8 concentrations, years of smoking and the male sex. 

Based on the Kotka’s cohort data, smoking is one significant risk factor for initial periodontitis, which modifies the oral microbiome to be more pathogenic, increasing dysbiosis and thus contributing to the onset and progression of initial periodontitis. The aim of the further examination of the Kotka cohorts data [29] was to investigate the possible risk factors and diagnostics of initial periodontitis in adolescents in the Kotka datasets, and to seek validation of preliminary previous results on the benefits of the aMMP-8 mouth rinse chairside test in identifying adolescents at risk of initial periodontitis. The identification of these risk factors in addition to diagnostics as well as individual preventive and early care should be centralized. Periodontitis can begin as early as in adolescence, but it has been observed that susceptibility to the onset and progression of periodontitis persists throughout life [12]. An incidence of severe periodontitis increases significantly after the age of 20 [5,30,31,32]. An evaluation of these periodontal outcomes is of interest to encourage oral health care prevention as implant-prosthetic rehabilitation may also be more at risk in a group of patients with a periodontal history [33]. Although tooth decay has decreased in young age groups (0–17 years), the same tendency has not been observed in periodontal diseases [34,35,36]. 

With the help of our studies on Finnish adolescents, we have tested the use of the aMMP-8 chairside test to detect early onset periodontitis in adolescents to support diagnostics [23,26]. With the aMMP-8 chairside test, it is possible to easily identify non-invasive adolescents at risk of periodontitis within 5 min. The test result can also motivate the adolescents to improve self-care [23,26]. It is also noteworthy that, as part of health guidance, smoking cessation intervention and detoxification by oral health professionals appear to be able to increase smoking cessation in adolescents [4,37].

Based on our studies based on Finnish data, up to 40% of adolescents cannot be classified as periodontally healthy or sick (no gingivitis or stage I periodontitis) according to the new classification. This group is the so-called grey area (preperiodontitis or subclinical condition), in which case it may be possible to move from one condition to another, that is, towards periodontitis or health. It is also possible that the latest classification of periodontal diseases does not serve its purpose for young patients at the risk of periodontitis. Adolescents at risk of periodontitis need individual prevention and, if necessary, early treatment, aMMP-8 test negativity, (≤20 ng/mL) [26] should be sought, as it is a biomarker of gum or periodontal health [23,26,29].

Early identification of patients at risk of oral diseases, targeted, individualized health guidance and prevention to reduce restorative care require also a change in attitudes. Recommendations such as by American Academy of Pediatric Dentistry (2023) for clinicians can be used to identify and manage the risk of periodontitis in adolescents [38]. A new Finnish innovation, antibacterial and anti-inflammatory photodynamic double light therapy, has shown promise in preliminary studies in removing residual plaque (biofilm) and lowering aMMP-8 concentrations in oral fluids [39,40]. For photodynamic double light therapy and the aMMP-8 mouth rinse chairside test, randomized controlled trials are needed to assess evidence-based efficacy in primary oral health care, including in adolescents. By shifting the focus from restorative care to prevention, we can also save money in the future, not to mention improve quality of life through improved oral health at all ages. The biggest and most significant work is done at home with nicotine-free, toothbrush, fluoride toothpaste and interdental cleaning equipment, independently.

## 5. Conclusions

It is important to allocate limited resources to preventing oral diseases among adolescents and strengthening self-care and better health behavior. Oral health care professionals can use the aMMP-8 mouth rinse chairside test and its results in early diagnosis of periodontitis, assessment of periodontitis risk and treatment outcome, while motivating their adolescents to improve oral hygiene. Oral health professionals also play a key role in quitting smoking among young people. The focus should therefore be shifted from restorative treatment to prevention, and resources should be allocated to this. In general, the prevention of periodontal disease should play a greater role in public health in adolescents and considering future potential cost savings in reducing the incidence of more severe periodontitis.

## Data Availability

Data are available on request from the authors. The data that support the findings of this study are available from the corresponding author upon reasonable request.
